# Direct visualization of chimeric antigen receptors on primary human T cells using *d*STORM super-resolution microscopy

**DOI:** 10.3389/fimmu.2025.1632823

**Published:** 2025-08-01

**Authors:** Leon Gehrke, Nicole Seifert, Peter Spieler, Christina Verbruggen, Rick Seifert, Fabio Toppeta, Maximilian Krick, Sören Doose, Hermann Einsele, Michael Hudecek, Markus Sauer, Thomas Nerreter

**Affiliations:** ^1^ Chair of Cellular Immunotherapy, Department of Internal Medicine II, University Hospital Würzburg (UKW), Würzburg, Germany; ^2^ Department of Biotechnology and Biophysics, Biocenter, Am Hubland, University of Würzburg, Würzburg, Germany; ^3^ Department of Internal Medicine II, University Hospital Würzburg (UKW), Würzburg, Germany; ^4^ Bavarian Cancer Research Center (BZKF), Würzburg, Germany; ^5^ National Center for Tumor Diseases (NCT), Würzburg, Germany

**Keywords:** CAR-T cell, immunotherapy, dSTORM, imaging, microscopy, CAR (chimeric antigen receptor)

## Abstract

Chimeric antigen receptor (CAR) T cells are a transformative treatment for hematological malignancies, and concerted efforts in the field are aiming to translate this success to solid tumors and autoimmune diseases. There is a desire in the field to accurately assess CAR organization and spatiotemporal expression to elucidate mechanistic details of CAR-T cell mediated anti-tumor activity and enable evaluation of the potency and safety of CAR-T cell products. We applied an IgG4-targeted F(ab)_2_ to achieve direct CAR labeling for super-resolution microscopy by *direct* stochastic optical reconstruction microscopy (*d*STORM). This enabled us to determine CAR surface expression on human primary T cells with single-molecule resolution independent of CAR specificity. We combined this direct CAR detection approach with a phenotypic assessment of the CAR-T cells, highlighting prospective applications to gain detailed mechanistic insights. With this new approach, we were able to detect the surface expression of CARs targeting SLAMF7, BCMA and CD19 with minimal background. We determined T cell subtype, donor material, and CAR construct as contributing factors shaping CAR surface expression and identified putative influence of CAR surface expression on CAR-T cell activation state. Here we provide a novel, tag-free approach to gain insights into the surface expression of CARs, illustrating the potential of super-resolution microscopy to inform the application of synthetic immune receptors for CAR-T cell therapy, potentially building the basis for more intricate and combinatorial studies to further improve the efficacy of CAR-T cell immunotherapy, predict therapeutic outcome and ensure optimal care for patients.

## 1 Introduction

Retargeting T cells by the introduction of chimeric antigen receptors (CARs), recognizing surface antigens in an MHC-independent manner, allows to specifically and efficiently combat cancer cells, and has proven to be a transformative treatment for hematological malignancies in recent years (1, 2). There are currently 7 CAR-T cell products approved by the FDA and EMA, accompanied by a large number of clinical and preclinical studies aiming to investigate the mode of action and the ideal product composition, to eventually transfer the success of CAR-T cells from hematological malignancies to solid tumor entities or even additional diseases like autoimmune disorders (3).

The main hurdles and therefore research foci in the field are limited efficacy in solid tumors, exhaustion-driven lack of long-term persistence of the CAR-T cells and (sub-)efficient migration to the target site. Another important aspect is the implementation of intrinsic safety mechanisms to counteract T cell toxicities associated with over-activation or on-target-off-tumor activity (4, 5). The CAR expressed on the cell surface is thereby a key mediator that governs antigen-dependent T cell signaling, determines antigen sensitivity, and initiates T cell cytotoxicity, proliferation and exhaustion (6). Upon antigen binding via the scFv, CARs utilize ITAM-based cytoplasmic motifs to initiate T cell activation via TCR-signaling-derived pathways (“signal 1”, 1^st^ generation CAR) in combination with co-stimulatory domains that harness additional signaling pathways to provide “signal 2” (2^nd^ generation CAR), and potentially induce activity of independent factors that modulate T cell activity (3^rd^/4^th^ generation) (6–8).

Though CARs enable T cell activation, thereby recapitulating TCR-mediated antigen recognition, they are not able to exploit the whole amplification capacity and sensitivity of the endogenous TCR signaling cascade. In contrast, they rather orchestrate the activation response to antigen binding in a more linear manner, translating the amount of antigen-bound CARs to signal intensity (9, 10). Certain CARs have been shown to exhibit a certain degree of tonic signaling, and some are actively engineered to mediate self-ligation/clustering to yield a moderate and beneficial baseline activation (11, 12). CAR expression levels have been linked to CAR-T cell performance and clinical outcome (13).

Therefore, detection of CAR-T cells and assessment of CAR expression in both, patient samples during clinical studies, as well as in preclinical studies, is crucial to enable accurate interpretation of CAR-T cell phenotype or therapeutic outcome and to gain mechanistic insights. Current approaches of CAR-T cell detection are commonly performed by using flow cytometry and indirect detection via transduction markers like LNGFR or EGFRt co-expressed with the CAR (14). Alternative approaches comprise direct labeling using purified target-antigen, or anti-scFv-specific antibodies (15, 16). In addition, tags like MYC or FLAG can be incorporated into the CAR itself in preclinical stages (17, 18).

Transduction markers, even though ideally transcribed and expressed concurrently with the CAR, cannot provide insights into actual CAR expression, and lead to over- or underestimation of the actual CAR expression due to different individual shuttling and recycling rates that result in disparate surface expression, rendering transduction markers a mere surrogate for actual CAR expression (14). CAR detection using the cognate antigen is a strategy that enables direct CAR labeling, and is commonly employed for clinical protocols (19). However, this is notoriously dependent on the scFv:antigen affinity, rendering comparison of binders targeting the same antigen with different affinities impracticable. Furthermore, this method is highly dependent on the availability of the target antigen as purified protein and its’ respective production cost, effectively limiting this approach to a small number of well-established targets and the respective CARs. The detection of the CAR via antibodies targeting the scFv eliminates the requirement of antigen production. However, it remains restricted to well-established CARs and is still subject to high costs without the benefit of comparing different scFvs side by side (20). While it is also possible to visualize the CAR with a fluorescent protein, this type of tag does not work in every CAR construct (21). Direct CAR detection can also be enabled using small molecular tags introduced directly into the CAR sequence. However, these have been reported to negatively influence CAR function and to carry a fundamental Host-versus-Graft (HvG) rejection risk, rendering them unsuitable for incorporation into clinical products (22).

Especially for settings of low CAR surface expression, it can be challenging to quantify the CAR using flow cytometry. Single-molecule localization microscopy (SMLM) offers single-molecule sensitivity and resolution beyond the diffraction limit, as demonstrated by various techniques such as *direct* stochastic optical reconstruction microscopy (*d*STORM), photoactivated localization microscopy (PALM) and DNA points accumulation for imaging in nanoscale topography (DNA-PAINT) (23–26). Recently these techniques, have been employed to resolve and quantify membrane receptors on the basal membrane of adhered cells (27–29). Here, we utilized *d*STORM to determine the CAR density. In this study, we applied an antibody targeting the CAR backbone in the IgG4 spacer/hinge region, which is used in investigational and approved CAR-T cell products, to enable scFv-specificity- and tag-independent detection via *d*STORM (30, 31).

## 2 Methods

### 2.1 Manufacturing of CAR-T cells

For the generation of human CAR-T cells, healthy donor peripheral blood mononuclear cells (PBMCs) were obtained from leucocyte reduction chambers provided by the Department for Transfusion Medicine of the University Hospital Würzburg. All donors provided written informed consent to participate in research protocols approved by the Institutional Review Board of the University of Würzburg.

### 2.2 Vector construction

epHIV7 lentiviral vectors containing CARs were constructed as previously described (32). To allow for specific selection and depletion of the generated CAR-T cells, all vectors additionally encoded a truncated epidermal growth factor (EGFRt) separated from the CAR transgene via a T2A ribosomal skip element sequence (14, 33).

### 2.3 Preparation of lentivirus for transduction

Lentiviral supernatants were generated using Lenti-X 293T cells (Takara, Japan), as previously described (34).

### 2.4 CAR-T cell generation

Human CAR-T cells were generated as previously described (32, 35).

### 2.5 Flow cytometry

Data was collected on a MACS Quant 10 (Miltenyi Biotech, Germany) and analyzed using FlowJo V10.8.1 (FlowJo LLC, USA). Cells were stained as previously described (35, 36). Antibodies used in this study were specific for murine IgG4 (Jackson Immuno Research, 115-006-006; polyclonal, cross-reactive to human IgG4), CD4 (Biolegend; 344612), CD8 (Biolegend; 344712), PD-1 (Biolegend; 367412), Tim-3 (Biolegend; 345026) Lag-3 (Miltenyi Biotech; 130-119-567) CD45RA (Biolegend; 304128), CD45RO (Biolegend; 304204), CD62L (Biolegend; 304806), EGFRt (Biolegend; 352906), CD25 (Biolegend; 302612), CD69 (Biolegend; 310928), and SLAMF7 (Miltenyi Biotech, 130-119-779), BCMA (Biolegend, 357506), CD19 (Biolegend, 982404) or the respective isotypes. Viability was assessed via 7-AAD (Miltenyi Biotech; 130-111-568), or Zombie aqua dye (Biolegend; 423102). T cell phenotypes were assessed as previously described (36, 37).

### 2.6 Functional assessment

For cytotoxicity assays, CAR or UTD T cells were co-cultured with ffLuc-transduced cell lines in medium supplemented with 150 µg/ml firefly D-luciferin (Biosynth, Switzerland; L-8220) at indicated effector:target (E:T)-ratios in technical triplicates. Tumor cell viability was determined by bioluminescence measurement on a Tecan Spark (Tecan, Switzerland). Specific lysis was calculated in reference to the corresponding UTD T cells.

### 2.7 Statistical analysis

Plots were generated, and statistical analysis was performed using Graphpad Prism V9.3.0. (GraphPad Software Inc., USA). Individual tests are indicated in the respective figure legend. P-values are stated exactly or represented by: **** = P ≤ 0,0001; ***= P ≤ 0,001; ** = P ≤ 0,01; * = P ≤ 0,05; ns = P > 0,05. CAR localization clusters/µm² are described as mean ± SEM in the text.

### 2.8 Cell lines and cell culture media

JeKo-1 and Raji (DSMZ, Germany), MM.1S and K562 (both ATCC, USA). TM-LCL feeder cells (provided by courtesy of Prof. S. Riddell (38), were maintained in RPMI-1640 medium (Thermo Fisher Scientific; 72400-054) containing 8% fetal calf serum (FCS), 2 mM L-glutamine (Thermo Fisher Scientific; 25030149), and 100 U/ml penicillin/streptomycin (Thermo Fisher Scientific; 15070063). Firefly luciferase (ffLuc)/GFP positive sublines and sublines expressing transgenes were generated by lentiviral transduction with the respective vectors at an MOI of 3 as previously described (39).

### 2.9 Cell staining for CAR detection by *d*STORM

5x10^5^ CAR-T cells were blocked with TruStain FcX (Biolegend; 422302) and stained with 2 - 15 µg/ml anti-IgG4 antibody (Jackson Immuno Research; 115-006-006) conjugated to Alexa Fluor 647 (Thermo Fisher Scientific; A20006) for 30 min on ice in FACS-Buffer or HBSS (Thermo Fisher Scientific, 14025-050). After staining, cells were washed, seeded in PBS on PLL (Sigma-Aldrich; P4707) coated (quantification experiments) or anti-CD45 antibody (Biolegend; 304002) coated (titration experiments) 8 well chambered cover glasses (Cellvis, C8-1.5H), fixated with 0.25% glutaraldehyde (Sigma-Adlrich; G5882) and 2% formaldehyde (Thermo Fisher Scientific; 28906) and washed with PBS (Sigma-Adlrich; D1408). TetraSpeck beads (Invitrogen, USA; T7279) were added in 80 mM Pipes buffer (Sigma-Adlrich; P1851) containing 1 mM MgCl2 (Sigma-Adlrich; PHR2486) and 1 mM EGTA (Sigma-Adlrich; 03777) at pH 6.8.

### 2.10 *d*STORM imaging

For reversible photo switching of Alexa Fluor 647 (Thermo Fisher Scientific; A20006), a PBS based imaging buffer (pH 7.4) containing 100 mM Cysteamine hydrochloride (Sigma-Adlrich, M6500-25G) was used. *d*STORM measurements were performed as previously described (24, 30). A 640 nm DPSS Laser (Novanta Photonics, USA; gem 640) was spectrally cleaned using a bandpass filter (Chroma, USA; Z640/10). The laser light was focused onto the back focal plane of an inverted Nikon Ti-E microscope, by two achromatic lenses (Thorlabs, USA; AC127-019-A-ML & AC508-150-A) mounted on a linear translation stage to adjust the TIRF angle. This microscope was equipped with an 100x, NA 1.49 TIRF-objective (Nikon, Japan; MRD01995) and a Nikon PFS to eliminate focus drift. Emission light was separated using a dichroic mirror (Semrock, USA; Di03-R405/488/532/635-t1) and filtered by a quadband filter (Semrock, FF01-446/510/581/703) and a long-pass filter (Chroma; ET 655 LP). The image plane was relayed by two identical achromatic lenses (Thorlabs; ACT508-200-B-ML) on a EMCCD camera (Andor Technology, UK; iXON DU-888U3-CSO-#BV), resulting in a pixel size of 129 nm. The entire system was controlled with NIS Elements AR (Nikon, version: 5.11.01). For each *d*STORM measurement, 15,000 frames were acquired with an integration time of 20 ms per frame at a laser power density of ~2.5 kW/cm². Alternatively, a commercially available ONI Nanoimager S (ONI, UK) was used to acquire 15,000 frames with an integration time of 10 ms per frame at a laser power density of ~3.5 kW/cm² and a pixel size of 117 nm.

### 2.11 *d*STORM data analysis

The raw data were reconstructed using rapidSTORM 3.3 (40). Further data analysis was conducted in python with LOCAN (41). Drift correction was performed with LOCAN using an iterative closest point (icp) algorithm or linear drift correction tool by rapidSTORM 3.3. The basal plasma membrane was segmented based on the reconstructed localization image and the corresponding brightfield image, to exclude signals of non-specifically bound antibodies on the coverslip. Localizations within the basal plasma membrane were clustered with a DBSCAN algorithm with the parameters ϵ = 20 nm and minimum points (MinPts) = 3. Cluster densities were estimated for the segmented plasma regions of interest. *d*STORM images were generated using LOCAN and napari, with an inferno color code representing the number of localizations (42). The localization precision was determined according to (43).

## 3 Results

**Figure 1:**
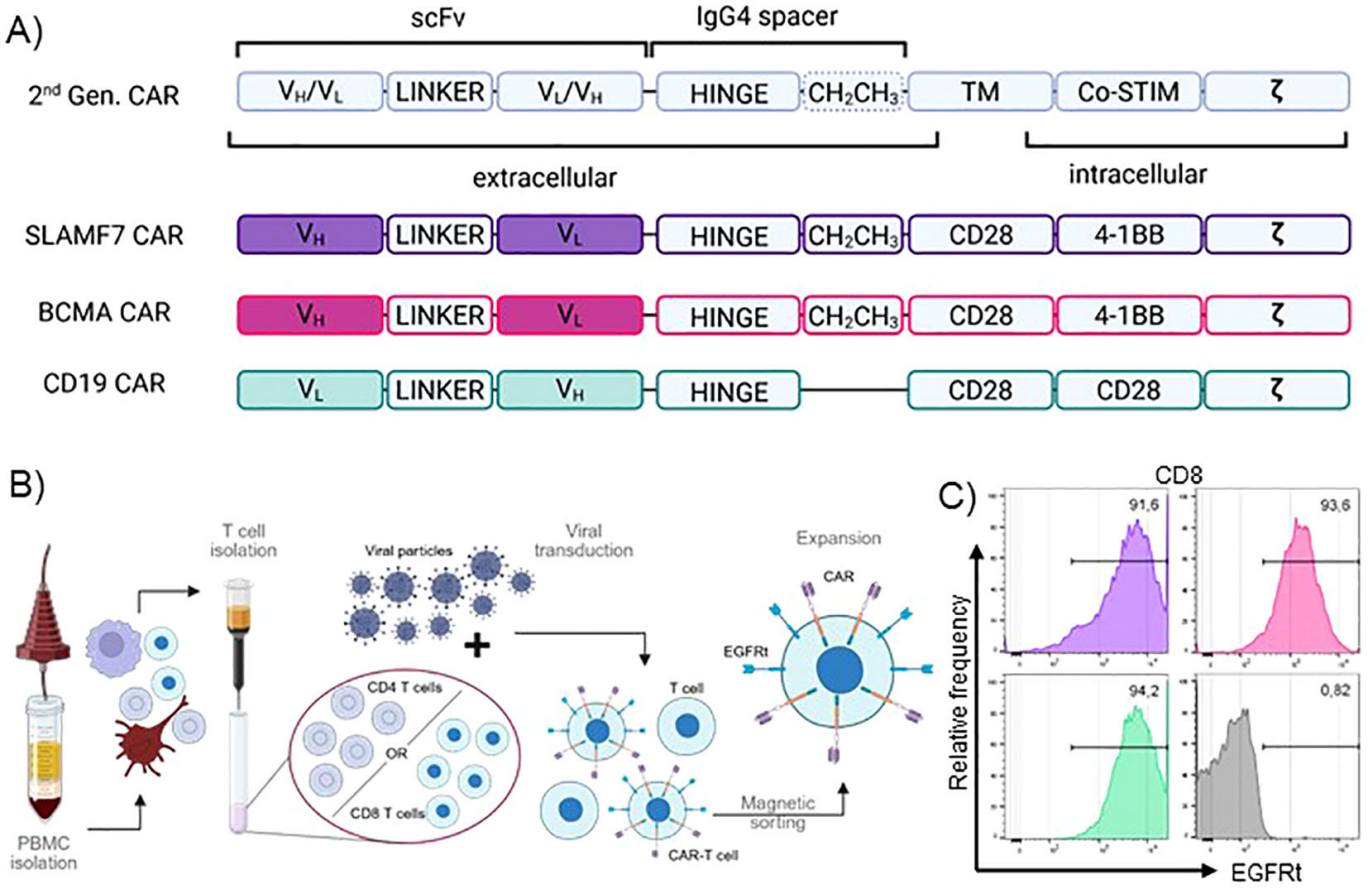
Production and quality control of pure IgG4-CAR-T cells from primary human PBMCs. **(A)** Schematic depiction of CAR design. TM, transmembrane domain; Co-STIM, Co-stimulatory domain; ζ, CD247 signaling domain; scFv, single chain variable fragment; VH, heavy chain variable domain; VL, light chain variable domain. **(B)** CAR-T cell production process. **(C)** EGFRt transduction marker expression of transduced, sorted and expanded CAR-T cells at post-production quality control. Representative plots show CD8^+^ T cells of one donor.

To achieve direct visualization of CARs in primary T cells and to show broad applicability, we used 2^nd^ generation CAR constructs targeting three different antigens relevant in hematological malignancies, which are either FDA-approved or under clinical investigation. All CAR constructs incorporate an IgG4-derived spacer domain to allow for subsequent visualization via antibody binding (**Figure 1A**). The CD19 CAR carries a version of the IgG4 spacer that lacks the additional CH2CH3 motif to investigate detectability of both IgG4 spacer variants prevalent in the field. The respective backbones were combined with scFvs targeting CD19, BCMA or SLAMF7. FMC63, binding to the paradigm CAR-target CD19, represents the best established and investigated scFv in CAR-T cell therapy, while B cell maturation antigen (BCMA) targeted via scFv BCMA30, is the most intensively-studied target in cellular immunotherapy of multiple myeloma (MM). In the approved products, BMCA is targeted, either via a scFv (Idecabtagen vicleucel) or camelid-derived nanobodies (Ciltacabtagen Autoleucel). The two well-established targets were accompanied by SLAMF7 with the scFv huLuc63 which is under clinical investigation as novel target in MM (44). All CAR constructs were accompanied by a surface transduction marker (EGFRt) separated by a T2A motif to ensure comparable expression rates. Using this library, we generated CAR-T cells from healthy donor material via viral transduction (**Figure 1B**). EGFRt-dependent magnetic sorting was used to ensure highly pure CAR-T cell populations, which were then subjected to the subsequent analyses (**Figures 1B, C**).

**Figure 2:**
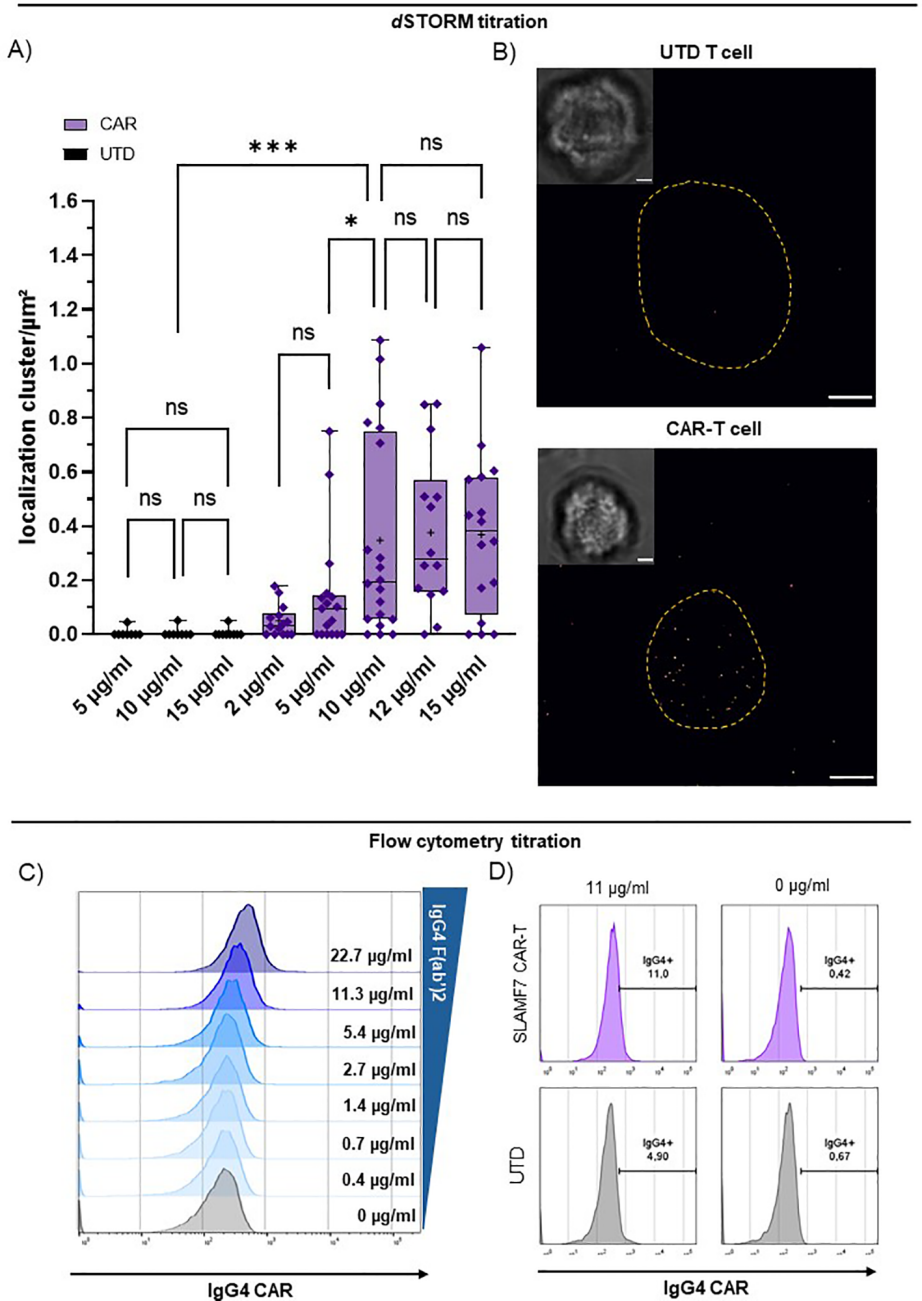
*d*STORM super-resolution microscopy achieves direct quantification of CARs via IgG4 F(ab’)2. Titration of CAR detection by anti-IgG4 F(ab’)2 fragment on CD8^+^ SLAMF7 CAR-T cells and UTD-T cells using flow cytometry and *d*STORM. **(A)** IgG4 F(ab’)2 titration for CAR detection in *d*STORM super-resolution microscopy. UTD and CAR-T cells were stained with increasing concentration of reagent. “+” indicates the mean; boxed-line indicates the median. Statistical analysis was performed using Brown-Forsythe and Welch ANOVA with Welch correction. **(B)** Representative bright field and *d*STORM images of UTD and CAR-T cell stained with the optimal concentration at 10 µg/ml IgG4 F(ab’)2. Scale bar = 2 µm. **(C)** Titration of commercially available FITC-labelled IgG4 F(ab’)2 for CAR detection via flow cytometry. **(D)** Representative flow cytometry plots of UTD and highly pure EGFRt^+^ CAR-T cells (compare: **Figure 1C**) stained with 11 or 0 µg/ml commercially available FITC-labelled IgG4 F(ab’)2. Significance indicated as: ***= P ≤ 0,001; * = P ≤ 0,05; ns = P > 0,05.

Next, we employed an anti-IgG4 F(ab’)2 antibody to target the CARs via the IgG4-derived motif (**Figure 1A**) enabling CAR detection via flow cytometry and super-resolution microscopy in combination with a cluster analysis (**Supplementary Figure 1**). Titration of the F(ab’)2 yielded clear detection and saturation with minimal background for *d*STORM (**Figures 2A, B**). We determined 10 µg/ml as ideal staining concentration for the *d*STORM approach being the lowest tested concentration, for which the CAR condition is significantly distinct from the matching UTD condition, while showing no significant difference in CAR detection to higher staining concentrations and no significant background between UTD conditions (**Figure 2A**). For the flow cytometric application,we used and titrated the commercially available FITC-labelled variant of the anti-IgG4 F(ab’)2 (**Figure 2C**). Proper discrimination between CAR-transduced T cells and CAR-negative UTD T cells, was not possible without significant frequency overestimation effects using flow cytometry (**Figures 2C, D**). Flow cytometry optimization by increased washing and additional blocking agents did not result in improved discrimination. In contrast, transduced and UTD T cells could be clearly discriminated using *d*STORM even at comparably high antibody concentrations (**Figure 2A**).

**Figure 3:**
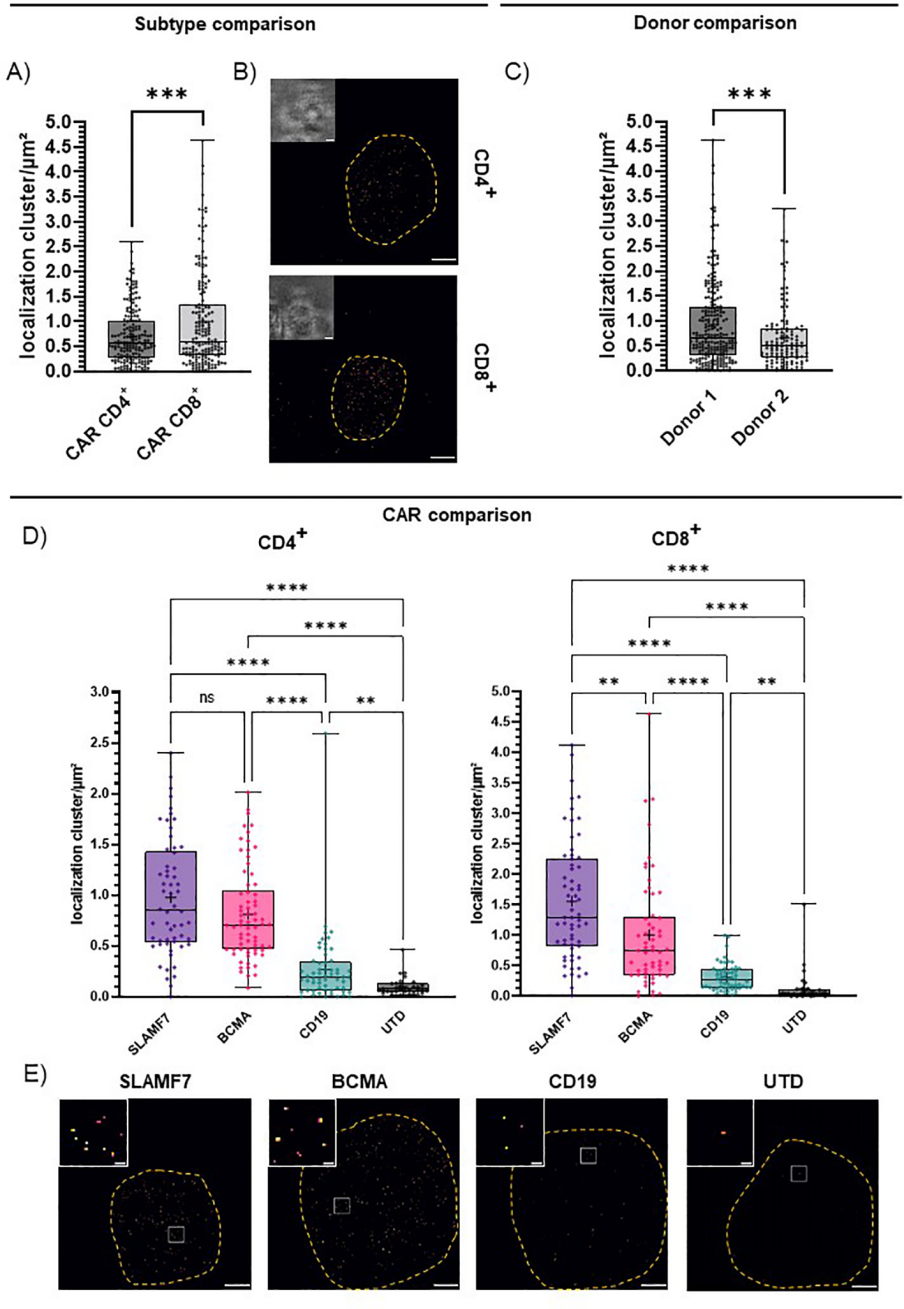
CAR surface expression is shaped by T cell subtype, CAR entity and donor. IgG4-based CAR detection by *d*STORM super-resolution microscopy via IgG4 F(ab’)2. **(A)** CAR expression of all CD4^+^ or CD8^+^ CAR-T cells throughout all CAR entities. Statistical analysis was performed using Welch’s t-test. CD4 (n=186), CD8 (n=176). **(B)** Representative bright field and *d*STORM images of CD4^+^ and CD8^+^ CAR-T cells stained with the optimal concentration at 10 µg/ml IgG4 F(ab’)2. **(C)** CAR expression of CD4^+^ and CD8^+^ CAR-T cells for two donors, throughout CAR entities. Statistical analysis was performed using Welch’s t-test. CD4^+^ donor 1 (n=133), donor 2 (n=53). CD8^+^ donor 1 (n=110), donor 2 (n=66). **(D)** CAR expression of distinct entities for CD4^+^ and CD8^+^ T cells throughout donors. Statistical analysis was performed using Brown-Forsythe and Welch ANOVA with Welch correction. CD4^+^: SLAMF7 (n=58), BCMA (n=70), CD19 (n=58), UTD (n=31); CD8^+^: SLAMF7 (n=64), BCMA (n=57), CD19 (n=56), UTD (n=32); “+” indicates the mean. The boxed line indicates the median. **(E)** Representative *d*STORM images (scale bar 2 µm) and cropped in images (scale bar 200 nm) of SLAMF7, BCMA, CD19 CD8^+^ CAR-T cells and CD8^+^ UTD T cells stained with ideal concentration at 10 µg/ml IgG4 F(ab’)2. Significance indicated as: **** = P ≤ 0,0001; ***= P ≤ 0,001; ** = P ≤ 0,01; ns = P > 0,05.

Utilizing single-molecule sensitive *d*STORM, we set out to assess the CAR surface expression and were able to elucidate factors that shape differences in CAR expression on the T cells already at baseline activation. First, we compared CD4^+^ and CD8^+^ T cell subtypes. Here, we could attribute a significant influence of the imaged T cell subtype on CAR surface expression even with all CAR entities pooled (**Figures 3A, B**). CD4^+^ T cells showed a lower CAR surface expression and a narrower range of expression levels throughout all specificities and donors (0.69 ± 0.04 localization clusters/µm²) compared to CD8^+^ T cells (0.98 ± 0.07 localization clusters/µm²; **Figures 3A, B**). We further compared two representative donors, to investigate inter-donor variation. We observed significant differences in average CAR surface expression in donor 1 (0.92 ± 0.05 localization clusters/µm²) and donor 2 (0.66 ± 0.06 localization clusters/µm²; **Figure 3C**). We further observed significant differences in CAR expression between the three selected CAR entities for both CD4^+^ and CD8^+^ CAR-T cells (**Figures 3D, E**). We detected a relatively high CAR surface expression for CD8^+^ SLAMF7 CAR-T cells (1.56 ± 0.12 localization clusters/µm²), intermediate levels for BCMA CAR-T cells (0.81 ± 0.05 localization clusters/µm²), low levels for CD19 CAR-T cells (0.31 ± 0.03 localization clusters/µm²) and negligible background detection for UTD T cells (0.13 ± 0.05 localization clusters/µm²; **Figure 3D**). In CD4^+^ T cells, differences in the detected CAR surface expression between the CAR entities were less pronounced, but the trends were similar. SLAMF7 CAR-T cells again showed the highest average surface expression levels (0.98 ± 0.08 localization clusters/µm²), closely followed by BCMA CAR-T cells with similarly high surface expression (0.81 ± 0.05 localization clusters/µm²). We detected the lowest receptor density for CD4^+^ CAR-T cells expressing the CD19 CAR comprising the CH2CH3 deletion (0.27 ± 0.05 localization clusters/µm²). We were not able to definitely rule out a lower detection due to potentially reduced binding of the shorter spacer. Still, given the suboptimal conditions this population could be significantly discriminated from background detection observed for CD4^+^ UTD T cells (0.10 ± 0.02 localization clusters/µm²; **Figure 3D**). In particular, the high detection sensitivity at very low surface densities, even under conditions of sub-optimal detection highlights the superiority of *d*STORM. While the mechanistic causes for the differences in detected CAR expression remain largely out of scope for the presented experimental setting, prospective studies that compare individual cases and specific therapy settings will elucidate these factors.

**Figure 4:**
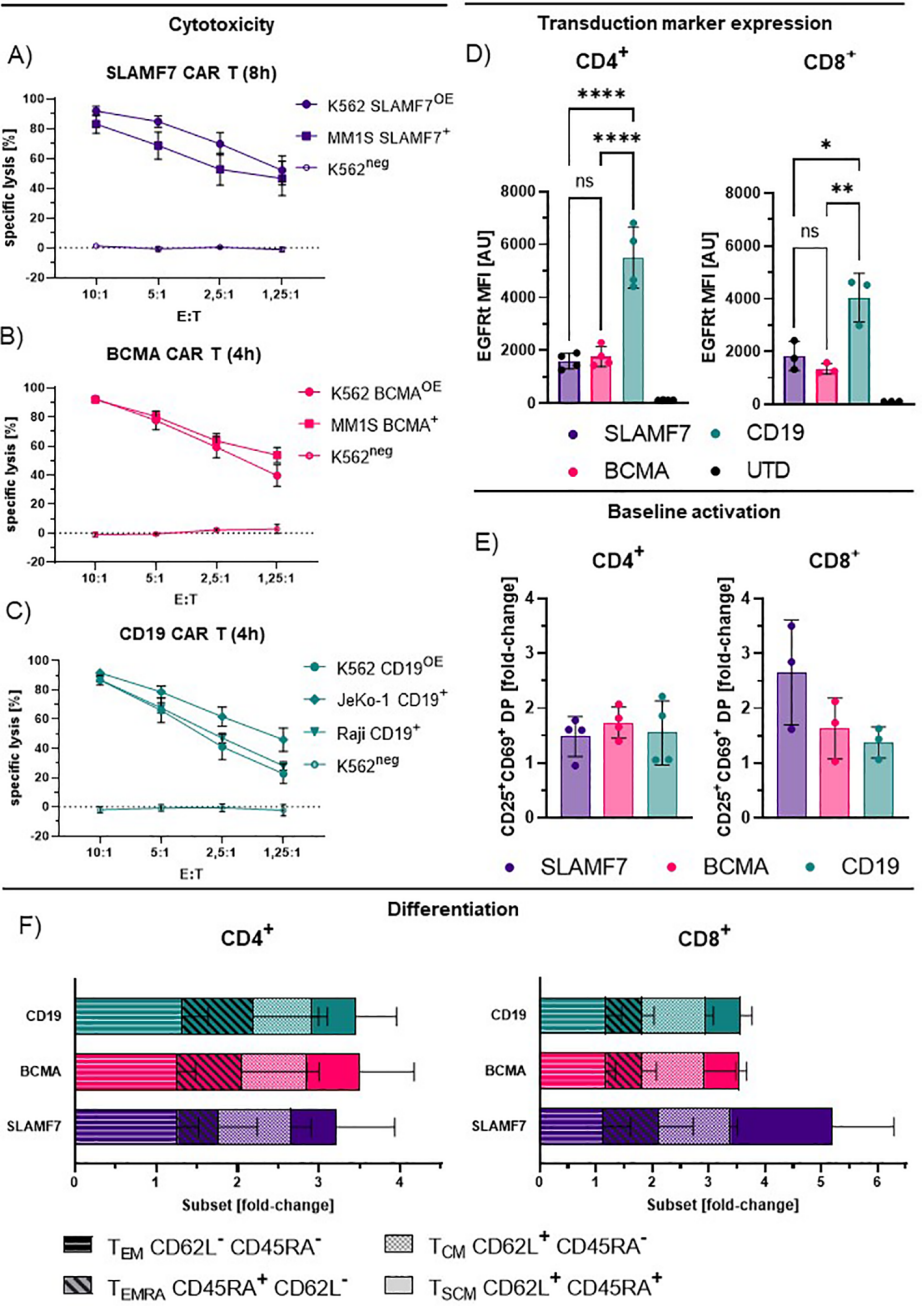
Functionality and phenotyping of IgG4-based CAR-T cells. **(A-C)** Specific lysis by CD8^+^ SLAMF7 **(A)**, BCMA **(B)** and CD19 **(C)** CAR-T cells incubated with the respective target cell lines for 4–8 h, shown for different effector to target (E:T) ratios. Values represent the mean +/- SEM of n=4. Endogenous expression (^+^), antigen over-expression (OE^+^), antigen-negative (neg). **(D)** Transduction marker expression for CD4^+^ and CD8^+^ T cells measured by flow cytometry. Values represent mean fluorescence intensity (MFI) +/- SD of n=4 (CD4^+^) and n=3 (CD8^+^). Statistical analysis was performed using one-way Anova for matched groups with Holm-Sidak’s correction **(E)** Baseline activation of CAR-T cells as fold-change of double-positivity (DP) rates of CD25^+^ and CD69^+^ cells compared to UTD. Values represent mean +/- SD of n=4 (CD4^+^) and n=3 (CD8^+^). **(F)** Relative frequency of CAR-T cell phenotypes for distinct CAR entities as fold-change compared to UTD; T_EM_, effector memory like; T_SCM_, stem cell memory like; T_CM_, central memory like; T_EMRA_, Terminal differentiated effector like. Values represent mean +/- SD of n=4. Significance indicated as: **** = P ≤ 0,0001; ** = P ≤ 0,01; * = P ≤ 0,05; ns = P > 0,05.

To investigate the effects of the distinct receptor densities detected via *d*STORM, we performed subsequent functional and phenotypic analysis. Here, all generated CAR-T cells exhibited specific and efficient cytotoxicity against established target cell lines expressing the respective antigen, independent of endogenous target expression, or ectopical overexpression after transduction of the respective target antigen (**Figures 4A-C**, **Supplementary Figure 2**). In contrast to the low CAR density detected for CD19 CAR-T cells, transduction marker expression intensity detected by flow cytometry indicates a high expression of the CD19CAR:EGFRt construct compared to the other CAR entities that match the respective *d*STORM data (**Figure 4D**). Thereby, suggesting a potential role of the CH2CH3 motif for efficient detection by the anti-IgG4 F(ab’)2.

In the absence of exogenous stimulus, all CAR-T cells showed increased frequencies of cells double positive (DP) for early and late activation markers CD69 and CD25, compared to donor-matched non-CAR-T cells (**Figure 4E**), suggesting a CAR-dependent baseline activation. CD4^+^ CAR-T cells did not show a significant difference in activation levels between CAR entities, despite pronounced differences in transduction marker expression intensity (**Figure 4D**, CD19) and CAR expression detected by *d*STORM (**Figure 3D**). Though, this is similarly true for the CD8^+^ CAR-T cells, here a trend is observable from high activation for SLAMF7 to lower activation for BCMA and CD19 CAR-T cells. This may be reflected by the link of SLAMF7 CAR-T cells to activation and fratricide mediated by self-expressed SLAMF7 on CD8^+^ (**Figure 4E**) (45, 46).

The observed differences in activation level between the CAR entities did not necessarily translate to significant differences in subtype distribution, though CAR introduction did induce expansion and contraction of subpopulations compared to non-CAR-T cells (**Figure 4F**). Effector-memory-like subtypes (T_EM_) were the most prominent phenotype, represented by around 70% of CAR-T cells throughout CAR entities and followed by central-memory-like T cells (T_CM_). Of note are the CD8 SLAMF7 CAR T cells as an outlier where higher activation coincided with pronounced expansion of the stem-cell-memory-like (T_SCM_) compartment.

Taken together, our data demonstrate that *d*STORM in combination with anti-IgG4 F(ab’)2 allows highly-sensitive detection of CARs and quantification of CAR density on human primary T cells. This technique is broadly applicable to IgG4 CARs even under suboptimal conditions and may be supplemented by functional and phenotypic analyses to provide the field with crucial mechanistic insights. In a first analysis we observed CAR expression to be construct- and cell type-, as well as donor material-dependent.

## 4 Discussion

In this study, we were able to detect and quantify CARs on the cell surface of primary human T cells, independent of CAR specificity with molecular resolution by *d*STORM imaging. CAR surface expression visualized using a broadly applicable anti-IgG4 F(ab’)2 fragment, differed markedly between conditions, and we identified donor material, T cell subtype, and CAR specificity, as significant factors shaping CAR surface expression (**Figure 3**). In a first analysis, we collected functional and phenotypic data that can inform CAR-T cell therapy approaches and enable mechanistic insights. Further, enabling prospective studies directly linking CAR density and CAR-T cell performance throughout CAR-formats and specificities.

We demonstrated that CAR detection via anti-IgG4 F(ab’)2 benefits from single-molecule detection sensitivity and low background signal levels to distinguish different expression levels, which were not reliably achieved using the commercially available antibody in flow cytometry. *d*STORM, a super-resolution microscopy technique, enabled high-precision measurements with minimal background to determine CAR expression levels.

Preclinical data involving the detection of CAR and CAR-T cells, which are used in the development of therapeutic regimens and advanced therapy medicinal products (ATMP), are affected by the way the CARs are visualized (19). As mentioned above, there are other methods that allow for direct labeling and detection of CARs, usually for a particular specificity, such as idiotypic antibodies and labeled cognate antigen or less commonly, for broader detection of multiple CAR specificities like Protein L or very recently an antibody targeting the GS4 linker (15, 16, 47, 48). The agent applied here represents a novel and valuable addition to the toolkit for CAR detection and can be adapted in laboratories without specialized infrastructure due to commercially available super-resolution microscopes (e.g. ONI Nanoimager S). Further, *d*STORM is not limited to this detection agent but many CAR designs will respectively be observable by introduction of established and new detection agents adapted to *d*STORM.

As shown, CAR expression differs markedly between different specificities. Previous reports have demonstrated expression level-linked effects on baseline activation by IgG4 CARs engineered for higher homo(di/multi)meric-interaction (49). Assuming, that higher frequencies of activated cells are derived from higher CAR expression, the *d*STORM-derived CAR surface expression data (**Figure 3D**) could match the rough trends in activation of the respective CD8^+^ CAR-T cells (**Figure 4E**). Though this particular experimental setting precludes direct comparison of CD19 and BCMA CAR-T cells due to potentially suboptimal detection conditions for CD19. In contrast to the other two CAR specificities, a higher degree of activation is apparent in SLAMF7 CD8^+^ CAR-T cells, which coincides with the highest CAR density. This may be an effect of the high CAR expression but could also be explained self-recognition and fratricide-linked activation via endogenous SLAMF7, and SLAMF7 expression has been reported to be higher on CD8^+^ than on CD4^+^ T cells (45, 46). The pronounced role of T_EM_ seen throughout CAR entities, may be caused by overlying expansion-induced stimulation effects that shape the phenotype of CAR-T cells, may interfere with resolving the purely CAR-induced effects but could be excluded using less stimulated cells and limiting expansion.

Previous studies have shown correlations between CAR expression, CAR-T cell performance, and clinical outcome for individual CAR constructs, highlighting the importance of exact CAR surface expression detection (13). In contrast to our approach, these studies were inherently unable to compare different CARs, or could not distinguish between baseline activation without stimulus and target binding-induced activation effects, thereby preventing a detailed comparison between different CARs and target.

An important parameter left out in many of the current state-of-the-art CAR-T cell studies is the exact antigen density that the respective CAR-T cells are engaging; although initial steps are being taken (27). The CAR-T cells proved to be functional and efficient in target cell killing, though the differences in antigen expression and the distinct susceptibility to CAR-T cell-mediated cytotoxicity of the target cells made a detailed comparison between the different CAR groups and mechanistic insights not feasible. While the present set up precludes further insights into the detailed relation between cytotoxicity and CAR vs target expression, *d*STORM is predestined for highly precise assessment of protein organization and antigen density measurements (27). The combination of precise antigen quantification on a specific cell line and highly sensitive CAR density measurement using *d*STORM on CAR-T cells can provide mechanistic insights into CAR-T cell function and the antigen levels required to elicit effective cytotoxicity. In addition, *d*STORM can reveal the upper and lower limits of CAR-density for CAR-T cell function, the ideal CAR format and scFv affinity/avidity for tumor entity-specific settings already early during preclinical testing if this link is made.

Since multiple CARs have been reported and engineered to facilitate homodimerization or clustering as well as heterodimerization, employing *d*STORM super-resolution with antibody derived staining agents allows for the assessment of clustering/dimerization events present at baseline or in the activated state (11, 49–51). Furthermore, it could give insights into the performance of distinct CAR constructs already at early stages of testing, as well as provide mechanistic insights into CAR organization signaling mechanisms, either comparing different specificities and CARs via the anti-IgG4 F(ab’)2, or single specificities in different CAR designs via scFv-based staining strategies in *d*STORM. These insights could be crucial, especially if linked to TCR signaling data and T cell functionality as already demonstrated partially in the presented analysis. This could be achieved in combination with other microscopy techniques like 3D lattice light-sheet TDI-DNA-PAINT to assess the resulting reorganization of the CAR surface distribution and improve the imaging of the quality of CAR synapse formation in the presence of cognate antigen. In addition, this may even allow for quantification of recruited surface proteins and respectively the induced signaling intensity (29, 52, 53).

In summary, we present here the first single-molecule sensitive, super-resolution quantification of CARs in the T cell membrane that is further, independent of their specificity and allows detailed comparison of the reciprocal, T cell-intrinsic and CAR- or binder-induced effects on CAR surface expression informing future more sophisticated studies of preclinical and clinical CAR-T cell products alike.

## Data Availability

The original contributions presented in the study are included in the article/**Supplementary Material**. Further inquiries can be directed to the corresponding authors.
