# Supplementary material for: Direct visualization of chimeric antigen receptors on primary human T cells using dSTORM super-resolution microscopy
Source: Front Immunol. 2025 Aug 1;16:1632823. doi: 10.3389/fimmu.2025.1632823 (PMC12354347; doi:10.3389/fimmu.2025.1632823)
Supplement: Supplementary file 1 [file DataSheet1.pdf]

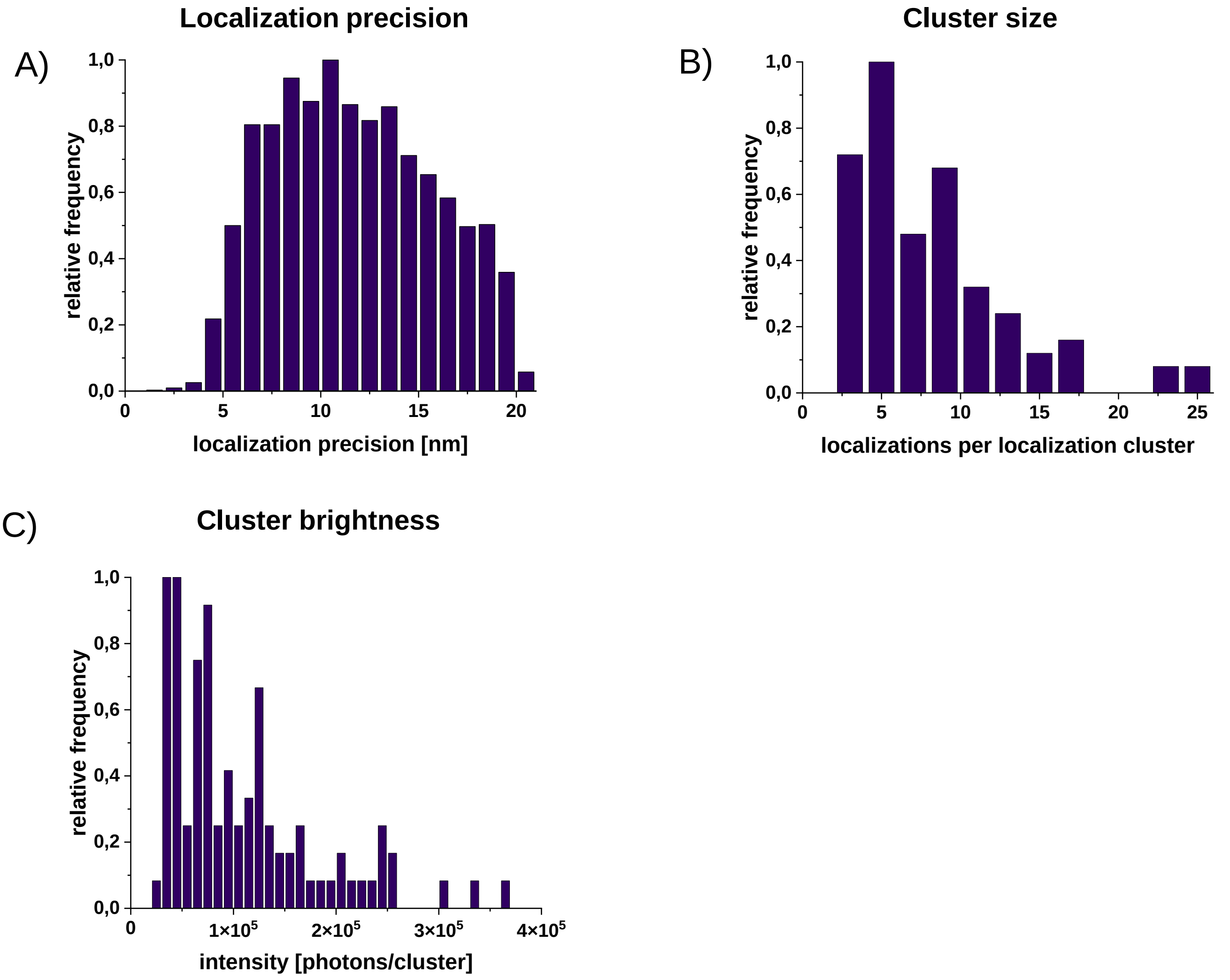

Supplementary figure 1: Localization cluster characteristics and localization precision.

Localization cluster characteristic and localization precision of SLAMF7 $CD8^+$ CAR-T cells from antibody titration. **A**) Localization precision of all selected ROIs from the antibody titration experiment. The average localization precision was 11.7 nm (+/- 4.1 nm SD). **B**) Number of localizations per localization cluster at the final antibody concentration of 10 µg/mL. The mean number of localizations per localization cluster was 7.7 (+/- 4.9 SD). **C**) Photon counts per localization for cells labelled with the final antibody concentration of 10 µg/mL**.** The average photon count was $1.1*10^5$ photons per localization (+/- $7.3*10^4$ SD).

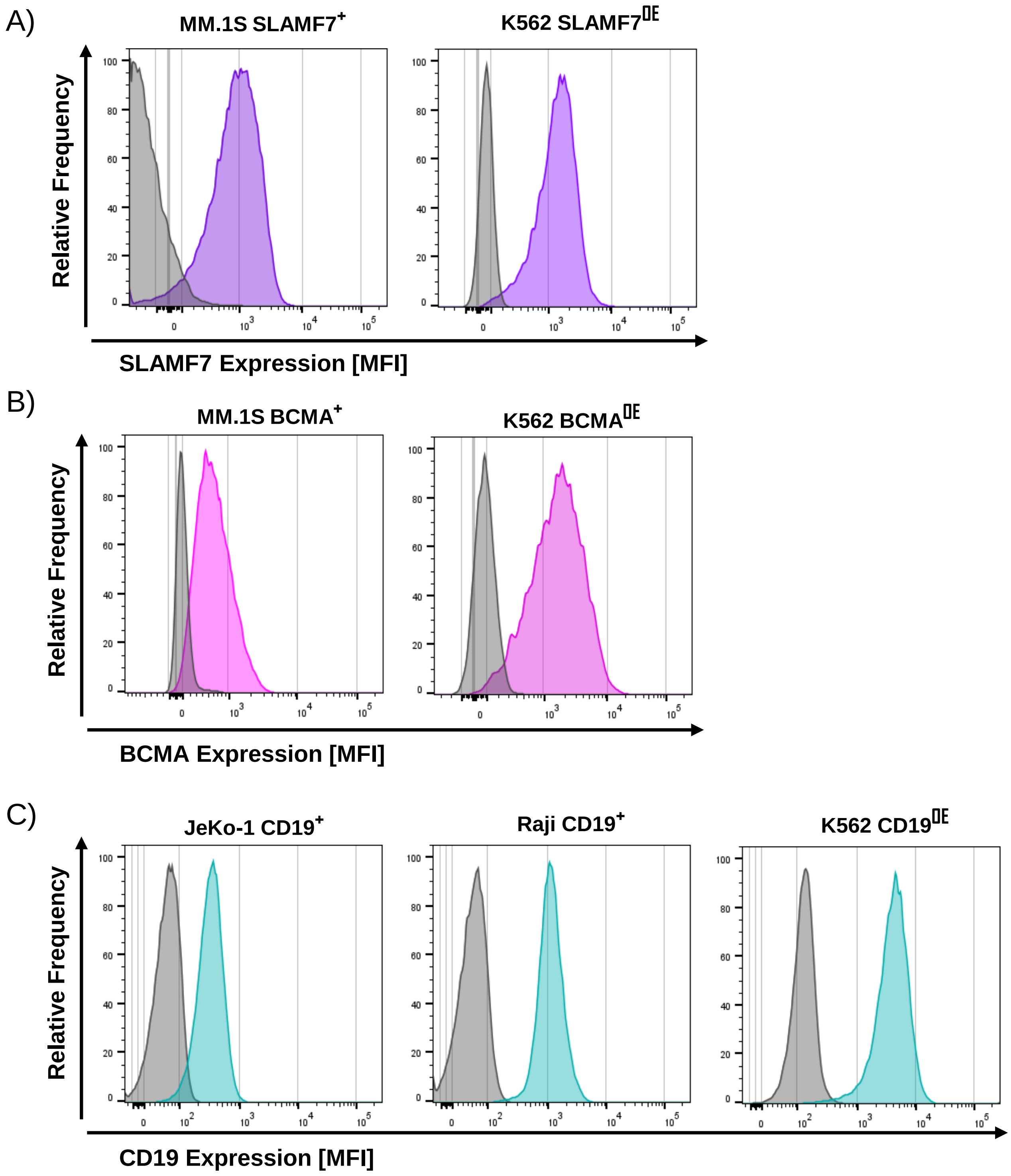

Supplementary figure 2: Target cell antigen expression.

A) SLAMF7 expression on MM.1S and K562 SLAMF7^{OE}, **B**) BCMA expression on MM.1S and K562 BCMA^{OE} and **C**) CD19 expression on JeKo-1, Raji and K562 CD19^{OE} measured by flow cytometry with isotype control.
